# Body Dimension Measurements of Qinchuan Cattle with Transfer Learning from LiDAR Sensing

**DOI:** 10.3390/s19225046

**Published:** 2019-11-19

**Authors:** Lvwen Huang, Han Guo, Qinqin Rao, Zixia Hou, Shuqin Li, Shicheng Qiu, Xinyun Fan, Hongyan Wang

**Affiliations:** 1 College of Information Engineering, Northwest A&F University, Yangling, Xianyang 712100, China; loraine@nwafu.edu.cn (H.G.); R-QinQ@nwafu.edu.cn (Q.R.); houzixia@nwafu.edu.cn (Z.H.); 1342524215@nwafu.edu.cn (S.Q.); 2 Key Laboratory of Agricultural Internet of Things, Ministry of Agriculture and Rural Affairs, Yangling, Xianyang 712100, China; 3 College of Computer Science, Wuhan University, Wuhan 430072, China; fxy.rebecca@163.com; 4 Western E-commerce Co., Ltd., Yinchuan 750004, China; nxwhy01@126.com

**Keywords:** transfer learning, deep learning, body dimensions, point cloud, Kd-network, feature recognition, FFPH, non-contact measurement

## Abstract

For the time-consuming and stressful body measuring task of Qinchuan cattle and farmers, the demand for the automatic measurement of body dimensions has become more and more urgent. It is necessary to explore automatic measurements with deep learning to improve breeding efficiency and promote the development of industry. In this paper, a novel approach to measuring the body dimensions of live Qinchuan cattle with on transfer learning is proposed. Deep learning of the Kd-network was trained with classical three-dimensional (3D) point cloud datasets (PCD) of the ShapeNet datasets. After a series of processes of PCD sensed by the light detection and ranging (LiDAR) sensor, the cattle silhouettes could be extracted, which after augmentation could be applied as an input layer to the Kd-network. With the output of a convolutional layer of the trained deep model, the output layer of the deep model could be applied to pre-train the full connection network. The TrAdaBoost algorithm was employed to transfer the pre-trained convolutional layer and full connection of the deep model. To classify and recognize the PCD of the cattle silhouette, the average accuracy rate after training with transfer learning could reach up to 93.6%. On the basis of silhouette extraction, the candidate region of the feature surface shape could be extracted with mean curvature and Gaussian curvature. After the computation of the FPFH (fast point feature histogram) of the surface shape, the center of the feature surface could be recognized and the body dimensions of the cattle could finally be calculated. The experimental results showed that the comprehensive error of body dimensions was close to 2%, which could provide a feasible approach to the non-contact observations of the bodies of large physique livestock without any human intervention.

## 1. Introduction

The availability of three-dimensional (3D) sensing models for large animals is becoming more and more significant in many different agricultural applications [1,2,3]. For the healthy cultivation and genetic breeding of large animals, periodic measurement of the animals’ body dimensions is necessary for breeders and researchers to master the growing complications related to pregnancy, laming, and animal diseases [4,5,6,7]. Large-scale measuring at a hold frame by skilled inspectors (traditional ways consist of a meter stick and metric tapes [8]) is ubiquitous, which has costs in terms of heavy manual labor, the animal’s stress response, and low efficiency due to long fatigue or differences in the individual experience among inspectors [9]. Very often, large animals cannot be effectively modeled on the spot by means of a series of classical 3D modeling methods due to their geometrical complexity or texture at growing periods, and 3D imaging or range scanners have been widely used to acquire the shape of an animal [10]. With the great achievements and breakthroughs of deep convolutional neural networks (DCNNs) in light detection and ranging (LiDAR) data classification [11,12,13], deep models could be reconstructed for the body measuring of large animals. This paper follows the state-of-the-art DCNN models and classical PCD processing methods, focusing particularly on the possibility of measuring the body dimensions of Qinchuan cattle, one of five most excellent beef breeds in China.

Qinchuan cattle are historic, famous, and have excellent performance in meat production. For an adult cow, the average height at the withers is about 132 cm and its average body weight reaches 420 kg, whilst for an adult bull, the average height at the withers is about 148 cm and its average body weight can reach above 820 kg. With the growing demand for meat from the vast population, the number of Qinchuan cattle is on the rise. To master the periodic feedback of growth and nutrition status in mass production, it is necessary to be able to automatically measure them in a feasible and effective way.

### 1.1. Livestock Body Measuring with LiDAR

With the development of 3D information technology, LiDAR (light detection and ranging) sensor scanning and structural light measurements have become important methods to quickly obtain 3D spatial data. They can be used to quickly and accurately obtain the coordinate data of the measured object surface and realize the non-contact measurement of the object. Compared with traditional measurement methods, 3D scanning measurement technology has many advantages such as fast speed, good real-time performance, and high precision [14]. According to different research objectives, relevant studies on the measurement requirements of 3D data in animal husbandry can be roughly divided into three categories: (i) to improve the breeding quality and precision feeding and to prevent diseases such as claudication; (ii) to analyze the body condition of specific parts; and (iii) to study the acquisition methods of body weight, body dimensions, etc. To generate a 3D cattle body model based on depth image, the point cloud data of cattle can be collected by a Kinect sensor to obtain 3D information of the cattle body to quantify the evaluation of cattle body condition [15]. By using the advantages of 3D depth image, researchers combined a thermal imaging camera and proposed a system for measuring the body shape and temperature of black cattle to complete the periodic quality assessment during the growth of cattle [16]. Some researchers have used a depth camera to obtain the body dimension data of pigs to estimate their weight, and found that the average absolute error of the nonlinear model measured by non-contact depth camera was 40% lower than that of the same nonlinear model measured by hand [17]. A dual web-camera high-resolution system was developed to obtain the 3D position of homologous points in a scene, which can be used to estimate the size and weight of live goats [18].

Due to the wide applications of livestock body measurement with 3D data, many processing and analysis methods have been proposed for different purposes. In order to measure pig body dimensions, the random sample consensus (RANSAC) algorithm can be used to remove the background point cloud and the foreground point cloud can be extracted with Euclidean clustering [19]. Additionally, some researchers have applied spatiotemporal interpolation techniques to remove moving noises in the depth images and then detect the standing-pigs with the undefined depth values around them [20]. Azzaaro et al. [21] used the linear and polynomial kernel principal component analysis to reconstruct shapes of cows with a linear combination of the basic shapes constructed from the example database and model validation showed that the polynomial model performed better than other state-of-the-art methods in estimating body condition score (BCS). These successful studies show that it is feasible to use 3D depth sensors to measure cattle body dimensions.

### 1.2. Applications of Deep Learning

In recent years, with sustainable development in the field of artificial intelligence and the continuous improvement of deep learning methods, image classification and recognition technology are developing toward a more intelligent direction. At present, image feature extraction is mainly divided into two categories: manual feature and mechanized feature extraction. Most representative and classical manual features are scale invariant feature transform (SIFT) [22], and histogram of oriented gradients (HOG) [23], and so on. These features are widely used for image classification of small datasets; however, in the case of large datasets, it is difficult to extract appropriate features from images [24]. Therefore, deep learning is commonly considered to extract high-level features of images to resolve this problem and reduce the impact of low-level features of manual feature extraction on image classification performance. At present, there have been many in-depth studies on two-dimensional (2D) image processing [25], and good results have been achieved in classification and recognition and other tasks. However, 3D data processing is still in its infancy. With the advent of 3D sensors such as IFM O3D303, Microsoft Kinect, Google Tango, and so on, 3D data have grown rapidly, and the recognition or classification based on 2D images has some limitations on special occasions. Image processing is developing from 2D to 3D, and the construction of deep learning networks for 3D image will be the hotspot of future research [26].

For 3D data sensed by LiDAR, deep learning techniques have been developed and applied for different occasions. To extract and classify tree species from mobile LiDAR data, deep learning techniques have been used to generate feature abstractions of the waveform representations of trees, which contribute to the improvement of classification accuracies of tree species [27]. A building detection approach based on deep learning utilizing the fusion of LiDAR data and orthophotos has been presented, and comparison experiments show that the proposed model outperforms the support vector machine (SVM) models in working area [28]. A way to segment individual maize from terrestrial LiDAR data has been proposed by combining deep leaning and regional growth algorithms, and it shows the possibility of solving the individual maize segmentation problem from LiDAR data through deep leaning [29]. 

The development of a neural network of 3D data has experienced three stages of PointNet [30], PointNet++ [31], and the Kd-network [32]. PointNet mainly resolved the problem of the disorder of PCD. The point cloud feature was abstracted point by point, and then the global feature vector was obtained by using symmetric function. PointNet++ has a hierarchical structure based on PointNet, divides more child point cloud in each level, and uses PointNet to extract the features of each point cloud. PointNet in local point cloud recognition has obvious advantages in accuracy, which is up to 95%. The wide applications of convolutional neural networks (CNNs) in 2D images provide the theoretical basis and technical support for the classification of PCD. A team designed and implemented a visualization technology to optimize the CNN model and deeply understand the functions and operations of the intermediate layers, which is the classic study of CNN visualization [33]. The Kd-network can perform fast computation through a 3D indexing structure, where the parameter sharing mechanism is applied and the representations from leaf nodes to the roots are calculated. However, this method needs to sample point clouds and to construct Kd-trees for every iteration and can employ multiple Kd-trees to represent a single object [34].

3D sensing for the non-contact measurement of body conditions has a good effect and effectively reduces the possibility of injuries to animal and inspectors [35]. With the development of deep learning models proposed for PCD, the evolving applications of PCD with deep learning could improve the accuracy of object classification and measurement, and similarly, the application of transfer learning could refine a deep model to an object. Transfer learning is an emerging research direction of machine learning, and unlike deep learning, it is mostly applied to cases of insufficient training data. The prior knowledge acquired by deep learning, namely the training results, can be applied to the relevant recognition fields. Most 3D data are not enough or perfect to satisfy all demands of different occasions and there also exists a lack of sufficient PCD of large livestock to train and verify deep models. Therefore, it is necessary to resolve the problem of the different distributions of the training and test dataset by means of transfer learning [36]. To improve the generalization ability of the deep model, the features and related examples could be transferred from a massive dataset to a trace dataset [37]. The deep model framework has a practical impact on the actual operation performance and classification accuracy. In this paper, the framework of PyTorch was employed after performance comparisons of the mainstream deep learning frameworks, whose application is more concise and flexible than the TensorFlow framework of PCD. 

### 1.3. Main Purposes

There are two key problems in realizing the automatic measurement of body dimensions of live Qinchuan cattle through transfer learning including how to preprocess the original PCD and how to transfer the features of PCD to obtain the target cattle body and how to automatically recognize the feature points of the body dimensions. For these problems, the original contributions of this paper can be summarized as follows:

- A new processing fusion for the 3D PCD of cattle is proposed. The original cattle PCD sensed by the LiDAR sensor was filtered by conditional, statistical, and voxel filtering, and then segmented by methods of Euclidean and RANSAC clustering. After the normalization of PCD and orientation correction of body shape, the fast point feature histogram (FPFH) was extracted to retrieve the body silhouettes and local surfaces.
- A 3D classification framework of the target cattle body based on transfer learning is presented. The PyTorch framework of the Kd-network was trained by the ShapeNet PCD dataset. The prior knowledge, the case-based transfer learning of the TrAdaBoost algorithm retrained by the collected cattle silhouettes, was applied to transfer the 3D silhouette of the point cloud and to classify the target cattle body point cloud. The PCD of the cattle body was normalized to extract the candidate surfaces of the feature points, and with extraction of FPFH, the feature points of the cattle body dimensions could be recognized.

The rest of this paper is organized as follows: Section 2 describes the proposed methods in detail. In Section 3, the automatic extraction experimentation of the feature points of body dimensions was carried out on the collected PCD of several groups of live Qinchuan cattle. Section 4 describes the experimental results. Section 5 discusses the performance of the cattle body dimension measurements. The last section summarizes the considerations, conclusions, and future works.

## 2. Materials and Methods 

**Figure 1:**
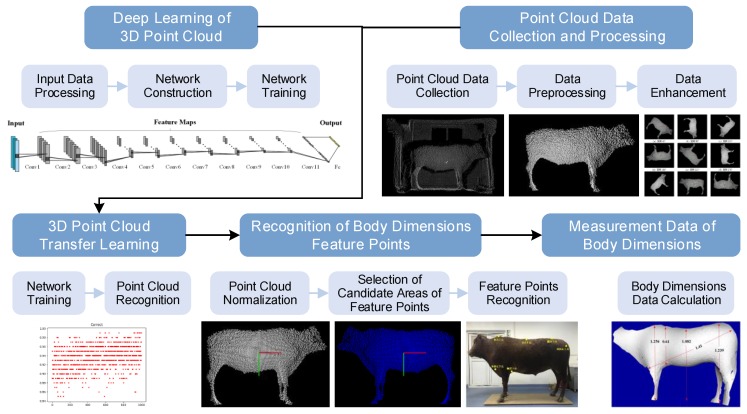
Flowchart of body dimensions measurement of Qinchuan cattle with transfer learning from LiDAR sensor.

An overview of the proposed methodology is shown in Figure 1, which combines the Conda software package management system under the PyTorch deep learning programming framework to develop the deep learning network in the environment of Python 3.6 and CUDA 9.1. The LiDAR sensor of IFM O3D303 (IFM Inc., Essen, Germany) was used to acquire the PCD of cattle body shape. Visual Studio 2015 was combined with the software development suite of IFM and Point Cloud Library (PCL), the C++ language was adopted to acquire and preprocess the collected PCD, and the acquired point cloud dataset of the cattle body shape was combined with the obtained deep learning network information for transfer learning. Finally, the recognition of the feature points of the cattle body dimensions was realized, and normalization of the cattle body point cloud and selection of the feature points were carried out to obtain the measurement data of cattle body dimensions.

### 2.1. D Point Cloud Deep Learning Network

The construction of a neural network is influenced by the defects of PCD collection and the characteristics of the point cloud itself. Three problems will be encountered in the acquisition process: data missing, noise, and rotation variability [38]. In addition, the characteristics of 3D PCD also affect the construction of a neural network including unstructured data, invariance of arrangement, and change in point cloud number [39].

To resolve the problems in the acquisition process and the point cloud’s own characteristics, in this paper, the Kd-tree network [40] was first used to make the 3D PCD have a fixed representation method and arrangement order. Then, the CNN was used to construct the deep learning network structure to realize the deep learning of the 3D point cloud.

**Figure 2:**
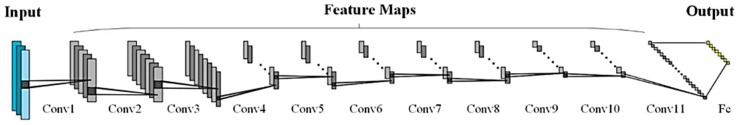
Network model hierarchy.

The size of the input PCD in this paper was $2^{11}$, and there were 16 categories. Therefore, 11 convolution layers were set, and each layer corresponded to each layer in the tree. The deep learning network framework was composed of 11 convolution layers and one full connection layer. The specific model hierarchy is shown in Figure 2.

Similar to the CNN, the network could share the weights in the same level, and used a bottom-up approach and the process was layered. In some sense, the representation of spatial positions on a certain layer was obtained through linear and nonlinear operations in the representation of multiple surrounding positions on the previous layer. In the same layer of the Kd-tree, the receptive field of any two nodes did not overlap [41].

Since the data of deep learning are not usually linearly distributed, the activation function needs to be used to add nonlinear factors [42]. Common activation functions include the Sigmoid function, Tanh function, and ReLU function. The ReLU function is the modified linear unit function, which is the mainstream neuron activation function in deep learning in recent years. Its mathematical expression is shown in Equation (1). When *x* > 0, the function gradient is identically equal to 1. Therefore, in the process of back propagation, the parameters of the first few layers of the network can be updated quickly to alleviate the problem of the disappearing gradient. Compared with the other two functions, the ReLU function is linear and unsaturated, which can significantly accelerate the convergence rate of the neural network [43].

(1) f(x)=max(0,x)

where x is the input neuron.

As there is no body shape dataset for animals to train the network, the 3D contour shape dataset of PCD in ShapeNet was selected. The training data contained 16 classes, a total of 15,990 samples of PCD files, which included planes (2421), bags (68), hats (49), cars (1641), chairs (3371), headsets (62), guitars (708), knives (352), lights (1391), laptops (400), motorcycles (181), cups (165), guns (247), rockets (59), skateboards (136), and tables (4739). (data source: [44]).

**Figure 3:**
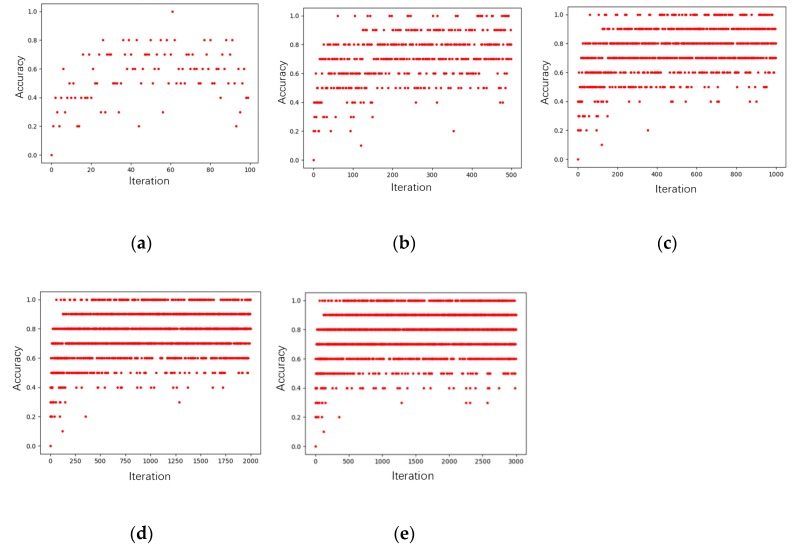
Accuracy rates with different training epochs. (**a**) Accuracy rates with 100 training epochs; (**b**) accuracy rates with 500 training epochs; (**c**) accuracy rates with 1000 training epochs; (**d**) accuracy rates with 2000 training epochs; (**e**) accuracy rates with 3000 training epochs.

Different training epochs have different influences on deep models. Smaller training epochs lead to under fitting and very large training errors. When the training epoch is increased to a certain number, the accuracy can basically remain unchanged or change a little bit with more and more computing facilities. If continuing to increase the epochs, it could waste more training time and more facilities [45]. The training epoch of 100, 500, 1000, 2000, and 3000, respectively, was selected to train the deep model, and the training results are shown in Figure 3, where the abscissa in each figure is the training epoch, and the ordinate is the corresponding accuracy rate. Table 1 is a comparison of the average accuracy rate with each training epoch. From Figure 3 and Table 1, as the training epoch increased, so did the accuracy rate. If the training epoch increases to or exceeds 1000, the changing tendency turns slowly. Considering the training consumption-time, learning rate, and computing loss, the training epoch was set as 1000 for this Kd-network model.

**Figure 4:**
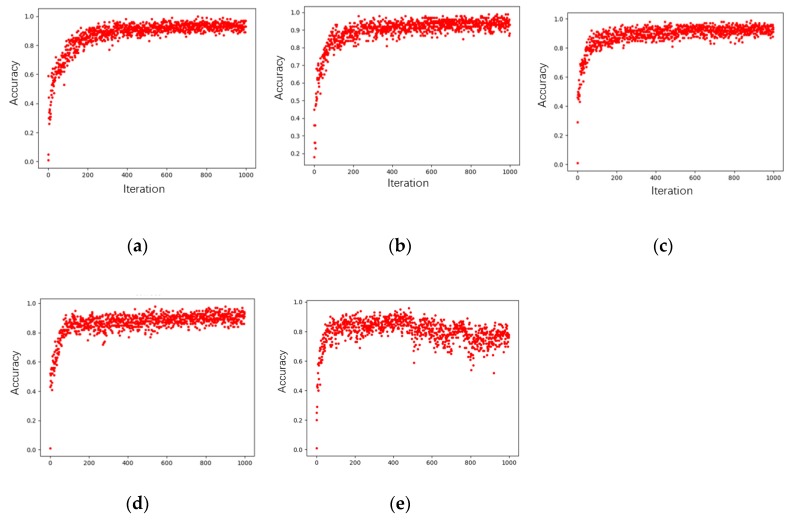
Accuracy rates with different learning rates. (**a**) Accuracy rates with a learning rate of 0.001; (**b**) accuracy rates with a learning rate of 0.003; (**c**) accuracy rates with a learning rate of 0.005; (**d**) accuracy rates with a learning rate of 0.007; (**e**) accuracy rates with a learning rate of 0.009.

The learning rate reflects the speed of gradient descent (SGD) in training, whose size can affect the convergence errors. If the learning rate is set too large, the error could be difficult to converge. If the learning rate is set too small, the consuming time could increase to cause the local optimization [46]. The learning rate of 0.001, 0.003, 0.005, 0.007, and 0.009, respectively, was set to train the deep network. The training results are shown in Figure 4, where the abscissa is the training epoch and the ordinate is the corresponding accuracy. It can also be seen from Table 2, that when the learning rate was less than 0.003, the average accuracy rate increased with the increase in the learning rate; when the learning rate was over 0.003, the average accuracy rate decreased with the increase in the learning rate. Considering the global SGD, computing facilities, accuracy, and number of samples, the learning rate can be set as 0.003 to achieve better training results. By selecting an appropriate training epoch and learning rate as above-mentioned, the comprehensive accuracy rate of 3D PCD could reach up to 89.6%, where the deep model can effectively extract, recognize, and classify 3D PCD.

### 2.2. Cattle Body Point Cloud Recognition Based on Transfer Learning

#### 2.2.1. Data Acquisition and Preprocessing

**Figure 5:**
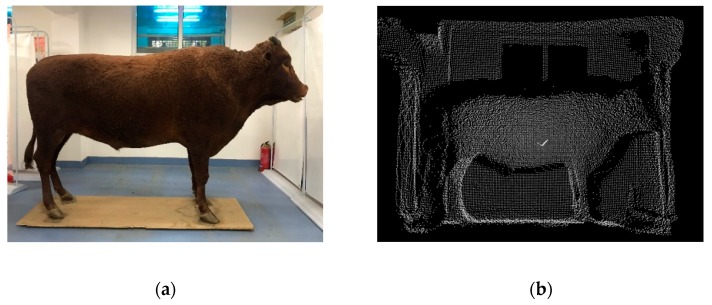
3D PCD (Point Cloud Data) acquisition for Qinchuan cattle (real specimen) with the LiDAR sensor where the 3D image shows the basic silhouette of the target cattle. (**a**) Shown in RGB; (**b**) Shown in 3D image.

The LiDAR sensor of IFM O3D303, based on the principle of Time of Flight (ToF), was employed to acquire the PCD of cattle shape. By sending continuous light pulses to the target, the sensor receives the light returned by the target, and calculates the flight time of the detected light pulses to obtain the distance of the target [47]. As the industrial depth sensor has relatively low resolutions, the captured PCD is sparse, therefore, every five frames, files could be stored to meet the body shape computation of enough density. To capture more concise and more precise PCD, the live cattle to be measured could be guided to be calm, or stationary or walk slowly in front of the LiDAR sensor by farmers or workers, and the cattle could be led to pass the measuring passage one by one, and not to trot or jump greatly. The original results of the 3D PCD are illustrated in Figure 5, where the 3D PCD after transformation clearly showed the basic contour of the target cattle with background information.

**Figure 6:**
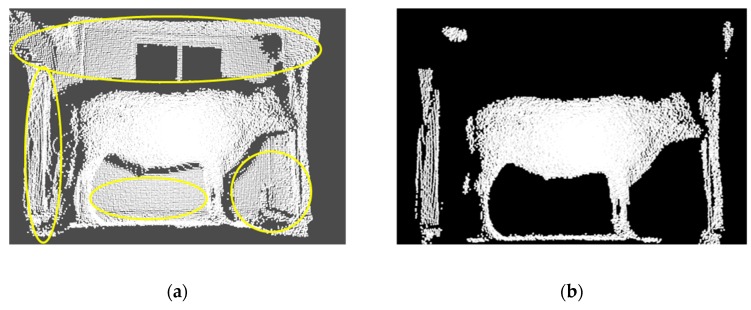
Filtering results with multiple filters. (**a**) Original PCD; (**b**) Results with three filters where most noises and outliers were well removed.

To obtain better results of segmentation, clustering, and feature extraction, the original PCD obtained needs to be preprocessed by a series of classical point cloud processing methods to cancel the background information such as different occlusions, target surface reflections, equipment errors, and so on [48]. According to the different characteristics of each filter, a preprocessing fusion with different filters was applied to achieve the best filtering results, where the conditional filter [49] was employed to remove the background with different distances, the statistical filter [50] to cancel outliers with different object surfaces, and the voxel filter [51] to compress the PCD of cattle. The fused processing results of multiple filters are shown in Figure 6, where much irrelevant information and outliers could be clearly eliminated. In Figure 6, the regions marked with yellow circles in the left were almost removed, leaving a clear PCD of the target cattle shape and other adherent outliers.

**Figure 7:**
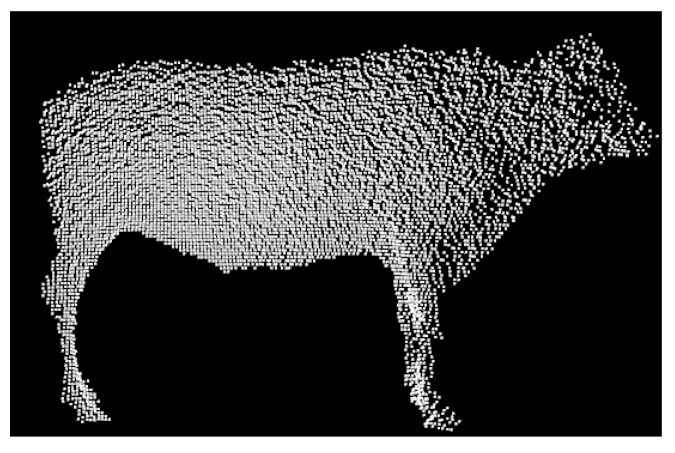
Segmentation with Euclidean clustering and RANSAC, where most of the background adherent to cattle has been canceled.

For some adherent outliers after preprocessing, the clustering segmentation [52] was required to obtain the PCD of a single cattle body. Based on Euclidean distance, the clustering segmentation [53] can only divide objects outside the target within a certain distance, and objects close to the target (such as the ground) can be classified into the same clusters, which cannot be separated [54]. To separate the same clusters of different objects, the RANSAC algorithm [55] is needed for plane extraction. The plane model is used to extract the ground PCD close to the cattle body and to segment a single file of cattle PCD. The segmentation results with Euclidean and RANSAC clustering are shown in Figure 7, where only a single cattle body silhouette is well preserved in the point cloud file.

Since there is no 3D PCD dataset for animals, all data need to be collected manually, so the amount of data obtained is limited [56]. However, a large amount of training data is needed in the training process of neural networks to prevent overfitting. Therefore, the affine transformation [57] was used to enrich the existing PCD of the cattle body silhouette.

By rotating PCD at different angles and by mirroring PCD in horizontal and vertical directions, the PCD of the cattle body can be enriched. As shown in Equation (2), the coordinate $\left(\begin{array}{ccc} x & y & z \end{array}\right)$ of PCD multiples the affine transformation matrix M to get a new coordinate of rotation $\left(\begin{array}{ccc} x^{\prime } & y^{\prime } & z^{\prime } \end{array}\right)$, where the rotation, mirroring, and other transformations can be realized.

(2) $$\left(\begin{array}{ccc} x^{\prime } & y^{\prime } & z^{\prime } \end{array}\right)=M\left(\begin{array}{ccc} x & y & z \end{array}\right)$$

**Figure 8:**
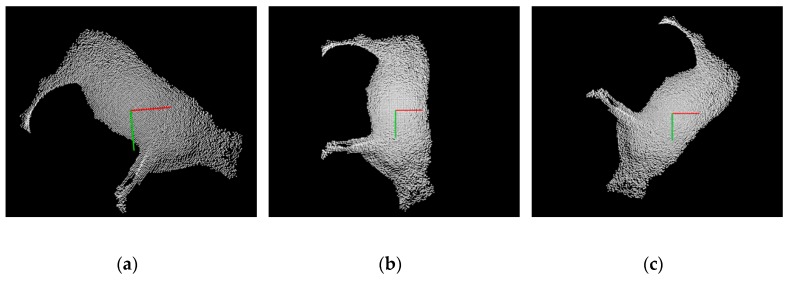

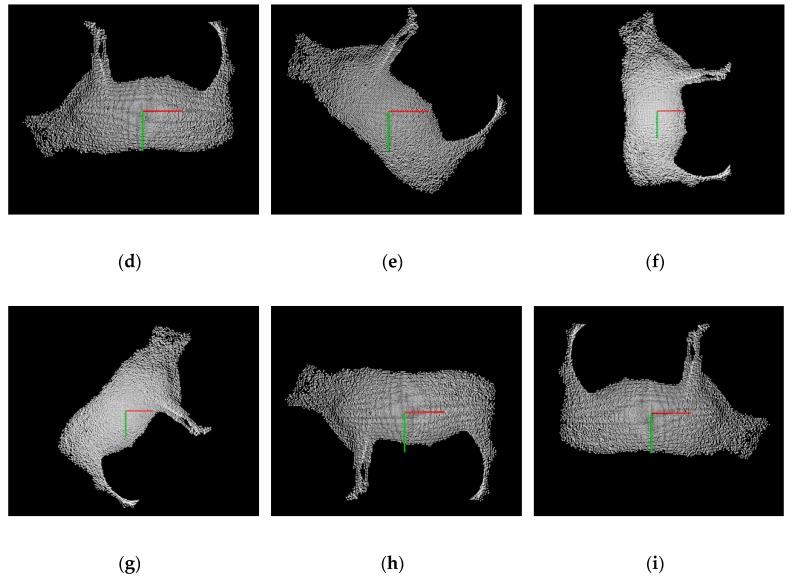
Transformation results of a single point cloud. (**a**) Rotation result with clockwise 45°; (**b**) rotation result with clockwise 90°; (**c**) rotation result with clockwise 135°; (**d**) rotation result with clockwise 180°; (**e**) rotation result with clockwise 225°; (**f**) rotation result with clockwise 270°; (**g**) rotation result with clockwise 315°; (**h**) horizontal mirror result; (**i**) vertical mirror result.

The PCD could be rotated clockwise along the 45°, 90°, 135°, 180°, 225°, 270°, and 315° as well as mirrored through horizontal and vertical transformation. The data augmentation operation expanded the original 251 cattle by nine times to a final dataset of 2510 PCD files of cattle bodies. The transformation results of a single PCD of the cattle body silhouette are shown in Figure 8.

#### 2.2.2. Design of Transfer Learning Network Structure

After the pre-training of the Kd-network, the initial parameters of the 3D deep model were obtained on large-scale ShapeNet network datasets. The spatial feature information of 3D PCD was extracted, and needed to be transferred to recognize the cattle body silhouette. For transfer learning here, the source data and the target data do not need to have the same data distribution [58].

In transfer learning, the PCD dataset of the cattle body was used as input to obtain partial output of the convolution layer that has been pre-trained on the Kd-network, and then use this output to train a fully connected network. Connect the convolution layer with the fully connected layer obtained from the previously pre-trained Kd-network, and then start the training of the transfer learning model. During transferring, there is training and verification in each iteration, and the loss can be back-propagated during training. Meanwhile, the parameters can be optimized, and the training results can be adjusted during verification. Each iteration, the loss value and correct value can be counted, the model with the highest correct value is saved, and then the next iteration of training is conducted. In transfer learning, the weights of all network layers, except the last fully connected layer, are frozen, and only the fully connected layer is modified so that the gradients during back propagation are not calculated, which can effectively avoid the occurrence of overfitting and improve training efficiency [59]. Finally, the original 16 outputs were changed into two outputs, that is, the original 16 classifications were changed into two classifications: cattle body point cloud and other point clouds.

In the training, 2510 enriched PCD of cattle body were applied for input, and the TrAdaboost algorithm [60] was used to transfer the samples. This algorithm can gradually improve the training weight of target samples in the examples of source datasets according to certain weight rules, reduce the weights of non-target samples, and improve the generalization ability of the model.

### 2.3. Recognition of Feature Points of Live Qinchuan Cattle Body

#### 2.3.1. Normalization of Cattle Body Point Cloud

In the process of collecting cattle data, due to the influence of the position and orientation of collecting equipment, the acquired PCD of the cattle body had a different orientation. To extract uniform features, the normalization method [61] was first used to calculate a unified orientation.

The standard measurement coordinate of cattle body was defined as follows: take the center of the mass of PCD of live Qinchuan cattle silhouettes as the coordinate origin, the body length direction of cattle as the x axis, the body height direction of cattle as the y axis, and the chest width direction of cattle as the z axis. The tail direction of the cattle is the positive direction of the x axis, and the direction pointing vertically to the ground is the positive direction of the y axis. The positive direction of three axes conforms to the right-handed rectangular coordinate system.

Principal component analysis (PCA) [62] was adopted to obtain the coordinate axis of the cattle point cloud and establish a new coordinate system. The processing flow is as follows:

(1) Obtain the center point of the cattle body.

Define the input point set of PCD of the cattle body $P=\left\{p_{i}|i=1,\;2,\;\cdots ,n\right\}$, where n is the number of points in the set. Then, the center point $p_{m}$ of the cattle body can be calculated by Equation (3).

(3) $$p_{m}=\frac{1}{n}\Sigma_{i=1}^{n}p_{i}$$

(2) Calculate the covariance matrix.

The covariance matrix $C_{p}$ of the PCD of cattle body can be calculated by Equation (4) with the center point $p_{m}$ of the cattle body.

(4) $$C_{p}=\frac{1}{n}\Sigma_{i=1}^{n}\left(p_{i}-p_{m}\right){\left(p_{i}-p_{m}\right)}^{T}$$

(3) PCA coordinate system is established according to feature vectors.

Three non-negative eigenvalues, $\lambda_{0}$, $\lambda_{1}$, and $\lambda_{2}$, can be obtained through the covariance matrix $C_{p}$, and Equation (5) is used to calculate the eigenvector $e_{0}$, $e_{1}$, and $e_{2}$, where $\lambda_{i}$ is the N-*the* eigenvalue of the covariance matrix $C_{p}$ and $e_{i}$ is the corresponding eigenvector of $\lambda_{i}$.

(5) $$C_{p}e_{i}=\lambda_{i}e_{i},\;i\in 0,\;1,\;2$$

The PCA coordinate system is established by taking the obtained center point $p_{m}$ of the cattle body as the coordinate origin, and the direction of the obtained characteristic vectors $e_{0}$, $e_{1}$, and $e_{2}$ as the direction of the x axis, y axis, and z axis, respectively.

The PCA coordinate obtained was ambiguous for the orientation of the cattle tail point to the positive direction of the x axis or the negative direction of the x axis. Here, the tail pointed uniformly to the positive direction of the x axis. The uniform orientation of all the PCD of the cattle body needed to be corrected as the highest point of the first half part of the cattle body was higher than that of the latter half part with common sense [63]. Define the point set $Q_{1}$ over zero in the point set of cattle P on the x axis after orientation correction, and define the point set $Q_{2}$ less than zero on the x axis.

$$\begin{array}{l} Q_{1}=\left\{p_{i}\in P|x_{i}>0\right\}\\ Q_{2}=\left\{p_{i}\in P|x_{i}<0\right\} \end{array}$$

The point sets $Q_{1}$ and $Q_{2}$ were searched to find the maximum points on the y axis direction, which were denoted as $q_{1}$ and $q_{2}$, respectively. If $q_{1}$ < $q_{2}$, the orientation of the cattle body is in the positive direction of the x axis and the point cloud of the cattle body needs to be corrected. Otherwise, no correction of the point cloud is required. A mirror transformation on point set P is needed and its symmetric data can be obtained by Equation (6) to change the orientation of the cattle body, where $\left(\begin{array}{ccc} x & y & z \end{array}\right)$ represents the coordinates before the mirror, and $\left(\begin{array}{ccc} x^{\prime } & y^{\prime } & z^{\prime } \end{array}\right)$ represents the new coordinates after the mirror. Finally, the tail of the cattle body in the corrected PCD files points to the positive direction of the x axis.

(6) $$\left(\begin{array}{ccc} x^{\prime } & y^{\prime } & z^{\prime } \end{array}\right)=\left(\begin{array}{ccc} x & y & z \end{array}\right)\left[\begin{array}{ccc} -1 & 0 & 0\\ 0 & 1 & 0\\ 0 & 0 & 1 \end{array}\right]$$

#### 2.3.2. Extraction of the Candidate Areas of Feature Points

To accelerate the selection efficiency of feature points in the PCD of cattle body, the candidate regions of feature points can be extracted. The curvature is the basic characteristics of the surface shape, reflecting the concave or convex degree of the PCD surface, and has a geometrical invariability of translation, rotation, scaling, and other transformation [64]. The mean curvature H [65] and Gaussian curvature K [66] were applied to extract the feature points in the candidate region of Qinchuan cattle.

It can be known from the differential geometry that there are countless normal planes at points in a surface, and normal curvature k is the curvature of the intersection line between the normal plane and the surface. The normal curvature k describes the curvature degree of the surface in a certain direction, and its maximum $k_{1}$ and minimum $k_{2}$ are the principal curvature of normal curvature. The normal curvature k in any direction can be calculated by the Euler equation, as shown in Equation (7), where θ is the angle between the osculating plane and the direction of the maximum value $k_{1}$.

(7) $$k=k_{1}\;{cos}^{2}\;\theta +k_{2}\;{sin}^{2}\;\theta$$

The mean curvature of a surface is the mean value of the two-principal curvature, as calculated by Equation (8), which can indicate the concavity and convex of a surface. Gaussian curvature K of a surface is the product of two principal curvatures, as shown in Equation (9), whose positive and negative value can determine the properties of points on the surface. When K>0, the points on the surface are elliptic points. When K=0, the points on the surface are parabolic points. When K<0, the points on the surface are hyperbolic points.

(8) $$H=\frac{1}{2}\left(k_{1}+k_{2}\right)$$

(9) $$K=k_{1}k_{2}$$

The principal curvature [67] of a point on a surface is the representation of the local shape of the point, which is independent of the surface parameters and can be calculated according to the first and second basic forms of the surface. Therefore, the mean curvature and Gaussian curvature can also be obtained by Equations (10) and (11), where $I_{x}$, $I_{y}$ are the first derivatives along the x axis and y axis, and $I_{xy}$, $I_{xx}$, and $I_{yy}$ are the corresponding second derivatives.

(10) $$H=\frac{\left(1+I_{x}^{2}\right)I_{yy}-2I_{x}I_{y}I_{xy}+\left(1+I_{y}^{2}\right)I_{xx}}{2{\left(1+I_{x}^{2}+I_{y}^{2}\right)}^{\frac{3}{2}}}$$

(11) $$K=\frac{I_{xx}I_{yy}-I_{xy}^{2}}{{\left(1+I_{x}^{2}+I_{y}^{2}\right)}^{2}}$$

Mean curvature and Gaussian curvature reflect the concavity and convexity of the surface, and nine kinds of combinations can be obtained according to the differences of positive or negative characteristics, as shown in Table 3. As the sixth kind of combination H=0 and K>0 is contradictory in math, it does not exist, so there are actually eight surface types that can be obtained by the combinations. The surface shapes of the corresponding combinations are shown on the right side of Table 3. Finally, through the calculation of mean curvature and Gaussian curvature, the cattle point cloud was classified according to the concavity and convexity of the feature points on the point cloud surface, and candidate areas of feature points were obtained.

#### 2.3.3. Feature Point Recognition

**Figure 9:**
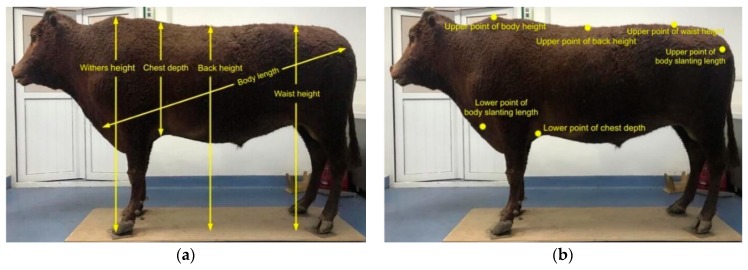
Schemes of adult Qinchuan cattle: (**a**) Five body dimensions; (**b**) Positions of feature points to be automatically acquired.

The measuring data of the body dimensions are the height at the withers, chest depth, back height, waist height, and body length, respectively, as shown in Figure 9. The points that need to be automatically obtained are the upper point of the height at the withers, the lower point of the cattle chest depth, the upper point of the height, the upper point of the waist height, the upper point of the body length, and the lower point of the body length, as shown in Figure 9b. As the height at the withers, chest depth, back height, or waist height is perpendicular to the ground, the lower point is the height at the withers or the lower point of the back height. The lower point of the waist height can be obtained by the y axis coordinate of the ground point as well as the x axis, the z axis coordinate of the corresponding point, and the upper point of the chest depth can be obtained through the x axis, the z axis coordinate of the lower point of the chest depth, and the highest point in the area regarded as the y axis. Finally, the fast feature point histogram (FPFH) [69] data were used to construct the feature model database of each cattle, and to recognize the feature point of the cattle body.

As the recognition process of each feature point is similar, we used the recognition of the upper point of the height at the withers as an example to describe it in detail. First, the FPFH values of the feature points in the candidate areas were calculated one by one according to the saving order, and then were matched with the values in the feature model database. If the difference between the two points is within a certain threshold, the matching is successful, and the point is judged to be the feature point of the height at the withers of the target cattle, and the search and calculation are stopped. Otherwise, match failure occurs and the same searching continues with the saving order until the match succeeds.

## 3. Results

**Figure 10:**
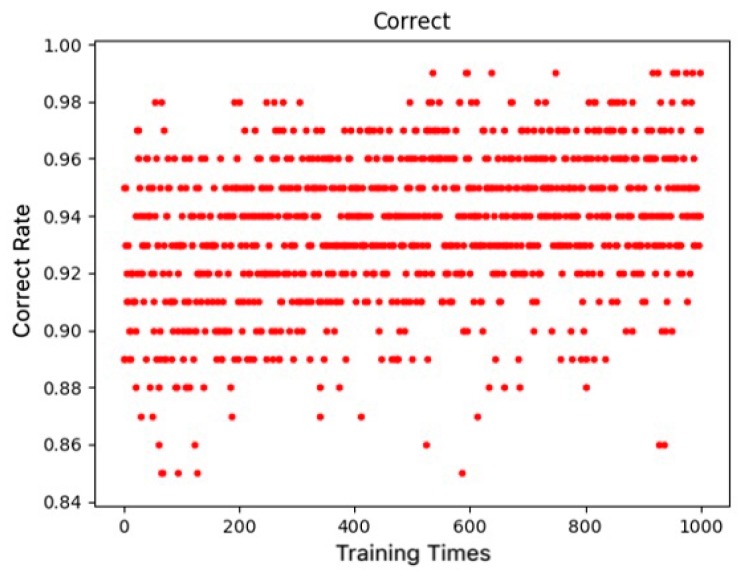
Correct rates of transformation training.

After the transfer learning model was trained, it took about 25 s to process the dataset and extract the relevant parameters. The non-contact measurements of the body dimensions were performed in Visual Studio 2015 with PCL 1.8 (CPU Intel i7 3.4 G-Eight cores, Gloway DDR4 RAM of 64 GB, Windows 10 of 64-bit, Nvidia GeForce GTX950 of 2G, CUDA 9.1). The running time of all the C++ code was less than five seconds from the acquisition, filtering, and clustering segmentation to reconstruction. Figure 10 shows the result of the transfer learning and training, with the abscissa representing the training epochs and the ordinate representing the corresponding accuracy rate. After training 1000 epochs, the accuracy rate of each training was calculated including all of the training epochs. The average accuracy rate of the deep model was 93.6%, which was greatly improved when compared with the previous average correct rate of deep learning.

**Figure 11:**
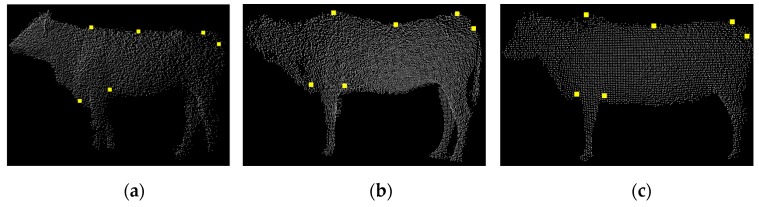
Recognition results of the feature points of three adult Qinchuan cattle: (**a**) Cattle Q0392; (**b**) Cattle Q0526; (**c**) Cattle Q0456.

**Figure 12:**
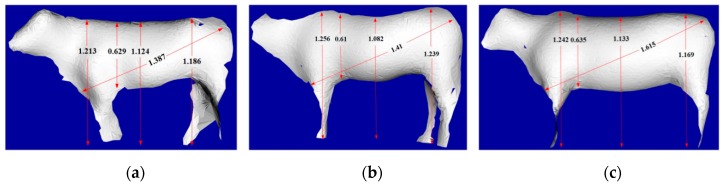
Automatic measurement results of the body dimensions of three live Qinchuan cattle: (**a**) Cattle Q0392; (**b**) Cattle Q0526; (**c**) Cattle Q0456.

To validate the deep model for measuring the body sizes of live Qinchuan cattle, the 3D PCD of three live Qinchuan cattle were collected at the National Beef Cattle Improvement Center, Ministry of Agriculture and Rural Affairs, Shaanxi Province. The ear tags of the three cattle were Q0392, Q0526, and Q0456, respectively. In the experimentation, six feature points of the body dimensions were extracted from the upper point of the height at the withers, the lower point of the chest depth, the upper point of the back height, the upper point of the waist height, the upper point of the body length, and the lower point of the body length. The visualization results of body dimensions feature points recognition of the three cattle are shown in Figure 11. Figure 12 shows the results calculated by automatically acquiring the feature points of the reconstructed cattle silhouette.

## 4. Discussion

The body dimension data of cattle Q0392, Q0526, and Q0456 extracted by automatic recognition and manual interactive extraction and their errors are shown in Table 4. The maximum error value of cattle Q0392 was body length, which was 2.36%, and caused by the large error during the recognition of feature points of the body length. For cattle Q0526, the chest depth data error value was the largest, which was 1.45%, while the height at the withers error value was the smallest, only 0.08%. For cattle Q0456, the error values of the height at the withers and chest depth was large, while the error of the back height was the smallest, only 0.09%.

From Table 4, the overall error of the body dimensions with the proposed deep learning model was within 0.03 m and the average error was about 0.02 m. In the calculated data of the cattle body dimensions, the error rate was basically within 1.5%. A bigger error in the body length of cattle Q0526 occurred through a bigger error of the upper point of the body length. The methodology can effectively achieve the automatic acquisition of feature point data of cattle body dimensions, avoid any manual intervention, simplify the measurement process, and improve the measurement efficiency.

## 5. Conclusions

This paper proposed an automatic acquisition of body dimensions with deep learning for live Qinchuan cattle. The PyTorch framework of deep learning of 3D point clouds based on a Kd-network was pre-trained by a 3D PCD dataset of ShapeNet. The training epoch and learning rate of the Kd-network deep model were analyzed with an average classification accuracy of 89.6%. After the 3D PCD acquisition of cattle by the LiDAR sensor, preprocessing of the classical filters fusion, and PCD processing of classical segmentation, the PCD of the cattle silhouette was extracted, and after data augmentation, the training dataset of the transfer learning based on the TrAdaBoost algorithm was created. The created dataset, as the target data, was applied to train the transfer learning model with a final average accuracy of 93.6%. After the normalization and orientation correction of the cattle body, the candidate feature regions were extracted with the mean curvature and Gaussian curvature. The FPFH of the candidate regions were extracted and matched. Finally, five linear body dimensions of cattle were calculated with the error rate within 2.36% and measuring errors within 0.03 m, and thus basically meets the measuring demands of real production.

The Qinchuan cattle is one of the five main beef cattle breeds in China, and the most widely used in northwest China. This method is only applicable to short-haired cattle breeds such as Qinchuan cattle and there have been proven large errors or even some big mistakes in our experiments with adult Qinghai yaks. Although some achievements have been made in theory, method and application, there are still many challenges to be resolved. In the near future, we will experiment with other beef breeds or crossbred cattle to validate these methods, which will improve the classification accuracy of the deep model by adjusting the network layers and designing convolution filters of different dimensions.

## Figures and Tables

**Table 1:** Comparison of the accuracy rate with different training times.

Training Epochs	Average Accuracy Rate
100	55.2%
500	69.8%
1000	76.1%
2000	76.8%
3000	77.3%

**Table 2:** Comparison of accuracy with different learning rates.

Learning Rate	Average Accuracy Rate
0.001	87.4%
0.003	89.6%
0.005	88.5%
0.007	86.7%
0.009	79.9%

**Table 3:** Local surface types of points [68].

Combination	Mean Curvature *H*	Gaussian Curvature *K*	Surface Type	Surface Shape
1	<0	<0	Saddle valley	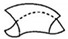
2	<0	=0	Valley	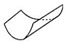
3	<0	>0	Well	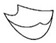
4	=0	=0	Plane	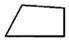
5	=0	>0	Does not exist	Does not exist
6	>0	<0	Saddle ridge	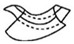
7	>0	=0	Ridge	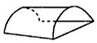
8	>0	>0	Peak	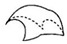

**Table 4:** The data and error values of the body dimensions of the three cattle (unit: m).

Ear Tag of Cattle	Data Extraction Method and Error	Withers Height	Chest Depth	Back Height	Waist Height	Body Length
Q0392	Automatic recognition	1.213	0.629	1.124	1.186	1.387
	Human-machine interaction	1.211	0.630	1.110	1.175	1.355
	Error value	0.17%	0.16%	1.26%	0.94%	2.36%
Q0526	Automatic recognition	1.256	0.610	1.082	1.239	1.410
	Human-machine interaction	1.255	0.619	1.095	1.237	1.414
	Error value	0.08%	1.45%	1.19%	0.16%	0.28%
Q0456	Automatic recognition	1.242	0.635	1.133	1.169	1.615
	Human-machine interaction	1.238	0.637	1.134	1.166	1.612
	Error value	0.32%	0.31%	0.09%	0.26%	0.19%
